# Exploring the well-being of professional female musicians: a self-determination theory perspective

**DOI:** 10.3389/fpsyg.2024.1465192

**Published:** 2025-01-06

**Authors:** Laurence Perrier, Laurence Latreille-Gagné, Florence Khoriaty, Maude Fortin, Arielle Bonneville-Roussy

**Affiliations:** Group of Research on Adulthood, the life Course, and Expertise (GRACE), Department of Psychology, Université du Québec à, Montréal, QC, Canada

**Keywords:** professional musicians, women, well-being, self-determination theory, qualitative interviews

## Abstract

**Background:**

This study investigated the well-being of 16 professional female musicians through the lens of Self-Determination Theory, focusing on the satisfaction of their psychological needs for autonomy, competence, and relatedness, as well as the unique challenges they encounter in their careers.

**Methods:**

Semi-structured interviews were undertaken and analyzed using thematic analysis.

**Results and discussion:**

Three broad themes and 10 sub-themes emerged from the interviews. The findings demonstrate that the well-being of female musicians is closely tied to the satisfaction of their psychological needs for autonomy, competence, and relatedness. Conversely, when these needs are frustrated, their well-being is negatively impacted. Other themes that emerged from the interviews are intrinsic motivation and the gender specific challenges within the music industry.

**Conclusion:**

The study highlights the need for supportive environments to enhance the well-being of female musicians (and performers as a whole), addressing both their psychological needs and the specific gender-related challenges they face.

## 1 Introduction

For over 20 years, the World Health Organization (2024) has identified psychological well-being as a pivotal health concern in contemporary society. Despite this recognition, well-being in performance domains, particularly music, has only recently garnered research attention (Ascenso et al., 2018; Bonneville-Roussy and Vallerand, 2020). Well-being generally refers to an individual’s overall mental, emotional, and social health, encompassing aspects such as life satisfaction, happiness, and the ability to manage stress and challenges (Seligman, 2018). The work of Ascenso et al. (2017, 2018, 2022) has been crucial in understanding how well-being is experienced in professional musicians. Their work has examined musicians’ psychological well-being through the PERMA model, which delineates five core elements of human flourishing: Positive Emotions, Engagement, Relationships, Meaning, and Accomplishment (Seligman, 2011).

Contrary to common perceptions of musicians’ lifestyles, Ascenso et al. (2017) observed that engaging in music is often viewed as challenging to well-being, noting that “Music activity has typically been considered a threat to holistic well-being” (p. 66). Their research, which involved in-depth interviews with professional musicians, revealed that some individuals struggle to separate their personal identity from their musical identity, leading to emotional and professional instability. However, the authors also found that many musicians demonstrate resilience: their ability to cope with and recover from the challenges they face in their careers. Professional musicians also experience flow, excitement, and positive emotions that enhance their overall well-being (Ascenso et al., 2017). Moreover, musicians often work in high-stress environments, where the pressure to constantly perform at a high level can lead to anxiety and a hindered sense of well-being (Bonneville-Roussy and Vallerand, 2020). In sum, well-being in musicians is multifaceted, involving the balance between managing the stressors of a high-pressure profession and nurturing the positive psychological satisfaction that comes with musical engagement. Furthermore, women in music face additional challenges, including longer leaves of absence, irregular schedules, and financial instability, which may hinder their well-being (Hruska and Bonneville-Roussy, 2022). Notably, in most countries worldwide, women continue to pursue gender equality in professional musical environments (Smith and Hendricks, 2022; Valenzuela et al., 2020).

Self-Determination Theory (SDT; Deci and Ryan, 1985) is a robust framework for examining motivation as a pathway to well-being, particularly in high-performance domains such as music. By focusing on the satisfaction of the psychological needs for autonomy, competence, and relatedness, SDT provides insights into how these factors contribute to overall well-being (Ryan and Deci, 2017). The present study aims to explore the well-being of female professional musicians through the lens of needs satisfaction and SDT. To explore these complex dynamics in professional female musicians, we employed a qualitative method, specifically thematic analysis of interviews, which are well-suited to capturing the depth and nuance of participants’ lived experiences (Braun and Clarke, 2006). Previous studies, such as Ascenso et al. (2017) have successfully used a similar methodology to uncover the multifaceted nature of musicians’ well-being. This approach enables us to align participants’ narratives with SDT’s theoretical constructs, offering a comprehensive understanding of the interplay between motivation, well-being, and gender-specific challenges in the music industry.

### 1.1 Self-determination theory and psychological needs

Understanding the mechanisms that underpin well-being in musicians is essential, particularly concerning how motivation can be sustained throughout their careers. The goal of nearly every classical music student is to become a professional performer. However, only a small percentage achieve this goal, with even fewer women reaching this level (Alberge, 2024). Those who achieve top-level success have invested thousands of hours in focused practice (e.g., Ericsson et al., 1993). Achieving such an elite musical status requires considerable motivation (Hatfield, 2024). One theory of human motivation frequently used to study musicians is SDT (Deci and Ryan, 1985; Ryan and Deci, 2017). SDT, as a theory of human motivation, focuses not only on the amount of motivation but mostly on the quality of that motivation. At its core, SDT distinguishes between intrinsic (driven by the sheer enjoyment and interest of doing an activity) and extrinsic forms of motivation (driven by internal or external outcomes, such as rewards or punishments), with intrinsic motivation fostering the highest quality of motivation. This theory has had profound implications in various fields, including music, where understanding the motivational dynamics can significantly influence career success among professional musicians (Evans, 2015; Evans and Ryan, 2022). For instance, Evans and Bonneville-Roussy (2016) and Bonneville-Roussy and Evans (2024) found that higher levels of autonomous motivation (in which intrinsic motivation is included) predicted greater practice frequency and higher levels of psychological well-being in student musicians. Most SDT research in music has focused on these types of motivation and how they relate to musicians’ achievement, persistence and well-being, especially in music students (Bonneville-Roussy et al., 2020; Evans, 2015; Evans and Bonneville-Roussy, 2016). This aspect may also be crucial for professional musicians, particularly in ensuring that their motivation is sustainable in the short term and throughout their musical lifespan (Evans, 2015) (Deci and Ryan, 1985; Ryan, 2023; Ryan and Deci, 2000, 2017).

SDT also posits that humans have three fundamental psychological needs—autonomy, relatedness, and competence (Deci and Ryan, 1985; Ryan, 2023; Ryan and Deci, 2017). The fulfillment of these needs fosters intrinsic motivation, growth, and well-being (Ryan and Deci, 2017). These needs align with key concepts of well-being, such as fostering positive emotions, engagement, and relationships (Seligman, 2011). While these three psychological needs are well understood in music students (Evans, 2015), their roles in the lives of professional musicians remain largely unexplored. SDT links the spectrum of extrinsic to intrinsic motivation with psychological needs by suggesting that the more the needs are satisfied within a given activity, the more intrinsically motivated a person is likely to be in that activity and the more they are likely to derive well-being from it (Cerasoli et al., 2016; Ryan, 2023; Ryan and Deci, 2017; Slemp et al., 2024). For elite performers, such as athletes, musicians, and experts in the workplace, satisfying these needs is critical to achieving peak performance and well-being. Nevertheless, most research on psychological needs satisfaction has been done with students or young athletes (Cerasoli et al., 2016).

### 1.2 Autonomy, competence, and relatedness in music

Autonomy refers to the need to feel in control of one’s actions and decisions. In the context of elite performance, autonomy is crucial for fostering intrinsic motivation. Studies have shown that when elite performers feel they have a choice in their decisions, they are more likely to feel intrinsically motivated and experience enjoyment, leading to enhanced performance (Bonneville-Roussy et al., 2020; Lonsdale et al., 2009; Lundqvist and Raglin, 2015). Moreover, autonomy-supportive environments that provide options and encourage input have been linked to higher satisfaction levels (Adie et al., 2012; Bonneville-Roussy et al., 2013, 2020).

Competence involves feeling effective and capable of achieving desired outcomes. For elite performers, continuous improvement and mastery are vital (Cerasoli et al., 2016). The sense of competence is reinforced through clear feedback and achievable challenge levels. In high-stakes environments, feedback that emphasizes skill development rather than outcomes helps sustain motivation in the long run. Research highlights that athletes who perceive their environments as supportive of skill development report higher levels of sustained engagement and performance (Cerasoli et al., 2016; Verner-Filion and Vallerand, 2018).

Relatedness is the need to feel connected to others. Fulfilling the need for relatedness can be particularly challenging for elite performers, including orchestral musicians. While orchestral musicians work in ensemble settings, the competitive and high-performance nature of their roles can still lead to a sense of isolation. Research has shown that a robust support system can significantly impact performers’ psychological well-being, particularly in long-cycle domains like orchestral music and competitive sports (Evans, 2015; Larson et al., 2019; Reis et al., 2000). For example, research by Williamon and Antonini Philippe (2020) has found that musicians and athletes both benefit significantly from a strong social support system to mitigate stress and enhance performance. Satisfying the basic psychological needs outlined by SDT is crucial for elite performers across various domains. Ensuring that these needs are met promotes higher performance levels and well-being.

Prior studies have underscored the critical role of fulfilling psychological needs in music. Freer and Evans (2019) have demonstrated that the satisfaction of the needs for autonomy, competence and relatedness directly influences the decision to continue in music. Research has shown that autonomy support is linked with musicians’ achievement, persistence and well-being (Bonneville-Roussy et al., 2013, 2020; Krause et al., 2019). Further supporting these findings, research has identified a positive relationship between fulfilling these needs and musicians’ persistence, higher levels of internal motivation, practice, and challenge-seeking. In contrast, frustration of these needs had the opposite effect, with less effective stress management skills and less ongoing commitment to a music career (Ascenso et al., 2017, 2018, 2022; McCready and Reid, 2007). Alessandri et al. (2020) have found that perceptions of competence among musicians are closely linked to their well-being. In professional musicians, creating a sense of belonging and feeling that they are part of a musical community is especially important. Although these studies mainly cater to music education contexts, they highlight outcomes relevant to the professional music setting. These findings collectively suggest that the satisfaction of the three basic psychological needs plays a pivotal role in the musical lives and well-being of musicians.

### 1.3 The challenges of being a woman in music performance

Women in the professional music industry face numerous challenges rooted in historical biases and contemporary societal norms (Smith and Hendricks, 2022). Research has shown that highly qualified music listeners are unable to distinguish between male and female performers when presented with auditory information alone (without visual information; Davidson and Edgar, 2003; Sergeant and Himonides, 2014). In spite of this, women remain disproportionately underrepresented in the classical music industry despite often having equal or greater representation in music education (Goldin and Rouse, 1997; Smith and Hendricks, 2022). Sergeant and Himonides (2023) have found that under 40% of musicians are women in the top 40 orchestras internationally. Men predominantly hold higher-level positions like conductors, soloists, principal musicians, and artistic directors (Scharff, 2018; Sergeant and Himonides, 2014, 2019). Women, on the other hand, are disproportionately more likely to become music teachers. While hiring practices have improved over recent years, discrepancies in gender pay persist (Smith and Hendricks, 2022). These structural challenges likely have significant psychological impacts on female musicians, yet their effects on well-being remain underexplored. While existing research has largely focused on issues such as representation and pay equity, it has often neglected the lived experiences of women in these professional contexts.

The primary challenge women face in Western classical music may no longer be overt sexism. Instead, a variety of factors may hinder the careers of female musicians. In a qualitative study that investigated the challenges of professional female musicians in Australia, Green and Mitchell (2023) have found that women face issues such as bias in the audition process, isolation, lack of support, stereotyping within male-dominated cultures, and a scarcity of female role models within orchestras. In addition, the greater caregiving responsibilities women typically assume may deter them from pursuing top-tier roles in the field (Hruska and Bonneville-Roussy, 2022). Moreover, women often encounter challenges related to work-life balance, particularly concerning ongoing family responsibilities. The need for touring and irregular hours can be particularly unforgiving for women seeking to balance family life (Cohen and Ginsborg, 2021). Many female musicians postpone or opt out of motherhood, fearing that taking parental leave might jeopardize their careers. Moreover, secure positions in music are scarce, with most being temporary contracts. This precarity is exacerbated for women returning from extended parental leave, who face the significant risk of being overlooked for rehire. Although women face these challenges, there is a lack of evidence of their impact on their daily lives and whether they affect their well-being.

#### 1.3.1 Needs satisfaction, well-being, and performance of female musicians

The intersection of gender and well-being in music performance has drawn increasing attention, particularly in understanding how female musicians experience stress, mental health, and career satisfaction. Research specific to female musicians indicates that they face unique challenges impacting their psychological health. For instance, studies have shown that female classical musicians experience poorer overall psychological health (Antonini Philippe et al., 2019). Similarly, Ascenso et al. (2018) found that female musicians report lower levels of positive emotions and overall well-being, especially at the beginning of their careers. Negative facets of well-being, such as depression, stress, and anxiety, are also reported mainly by women (Bonneville-Roussy et al., 2017; Loveday et al., 2023; Musgrave, 2023).

In broader research on needs satisfaction, findings have shown that autonomy tends to be lower in women, while relatedness and personal growth are higher, highlighting differing values that may shape career experiences (Matud et al., 2019). Additionally, job stress appears to affect women’s well-being significantly, with potential cumulative impacts over time, though social relationships at work can buffer the adverse effects of stress on their well-being, offering valuable sources of support and resilience (Mensah, 2021). Despite these insights, existing studies on the lived experiences of female musicians often focus on negative outcomes, such as stress or burnout, while neglecting the positive aspects of well-being, such as motivation, fulfillment, and personal growth. This imbalance highlights the need for a more comprehensive approach to studying female musicians’ well-being. By framing the research around SDT, this study aims to fill this gap by capturing both the challenges and sources of well-being in the professional lives of female musicians.

### 1.4 Research questions

The present study aims to answer these two research questions through the lens of SDT:

1. What factors positively and negatively relate to the well-being of female professional musicians?
2. What specific challenges do women face in the music industry?

A qualitative interview study was used to explore these questions. This method allowed for a detailed and nuanced understanding of the participants’ lived experiences, providing rich, contextual data that quantitative methods might not capture (Braun and Clarke, 2006).

## 2 Methods

### 2.1 Participants and procedure

As part of a larger research project involving two quantitative research surveys and an interview on the links between motivation, performance and well-being in high-achieving domains, such as sports, high-skilled careers and music, over 200 high-achieving individuals were recruited to take part in the quantitative section of the project, of which 16 female musicians agreed to participate in the qualitative interviews. The participants were recruited by word-of-mouth and snowballing in professional classical orchestras in Canada. Therefore, they were all professional, active classical musicians. The participants had a mean age of 40.44 years (25–58 years, SD = 10.84). Participants had, on average, 31.31 years of experience with their instrument. Their musical instruments included woodwind, brass, strings and keyboard. Descriptive statistics for each of the participants are found in Table 1.

**Table 1:** Descriptive statistics of each of the female participants included in the study.

Participant	Instrument family	Age	Playing years - professional	Playing years - in general	Level of education
Part. 1	Strings	58	40	53	Master’s
Part. 2	Strings	41	17	36	Master’s
Part. 3	Strings	58	35	55	Bachelor’s
Part. 4	Strings	57	28	–	Master’s
Part. 5	Keyboard	32	5	12	Doctorate
Part. 6	Woodwinds	45	23	40	Master’s
Part. 7	Strings	25	10	20	Bachelor’s
Part. 8	Strings	39	21	35	Doctorate
Part. 9	Strings	42	25	33	Master’s
Part. 10	Woodwinds	34	13	23	Master’s
Part. 11	Woodwinds	33	7	23	Master’s
Part. 12	Brass	34	10	17	Master’s
Part. 13	Woodwinds	33	16	26	Master’s
Part. 14	Strings	35	16	30	Doctorate
Part. 15	Keyboard	53	30	45	Doctorate
Part. 16	Woodwinds	28	10	25	Master’s

Two postgraduate students in music psychology interviewed the participants. The interviews lasted on average 37.25 min, and were recorded using the interviewers’ phones, then password-protected, anonymized, and transferred to a university-protected cloud account.

To minimize bias, the interviewers were trained to ask questions, prompts, and follow-up questions consistently across all study participants. One interviewer was a classical musician herself. Therefore, to minimize interviewers’ bias, we ensured that the researcher did not interview participants who had a working or close relationship with her.

Ethical approval was granted by the authors’ institution (2022-4224). All participants signed a consent form and were sent the interview questions beforehand.

#### 2.1.1 Interview questions

The interviews were targeted toward a specific activity, depending on the participant’s primary area of expertise (music, sports or work). Subsequently, the questions all referred to this activity (in the present study, music performance). At the beginning of the interview, participants were asked to name their area of expertise (in this case, music performance, but they had the opportunity to elaborate, for instance, by providing their musical instruments) and to refer to it when answering the questions. Since the interview was not targeted only toward musicians, the research team chose not to alter the questions to minimize bias. Participants were asked the following four questions in order:

1. How do you feel when you are doing your activity?
2. Does this activity give you a sense of well-being?
3. Does this activity have a negative impact on your well-being?
4. In general, does this activity have a positive or negative impact on other aspects of your life (social, leisure, work, family)?

### 2.2 Data analysis

The study employed an inductive-deductive thematic analysis following a structured methodological approach (Braun and Clarke, 2006; Fereday and Muir-Cochrane, 2006). Firstly, recorded interviews were transcribed verbatim by two interviewers and a research assistant. This technique was selected for its methodical organization of qualitative data. The study initially used deductive thematic analysis by drawing on the established theoretical frameworks of SDT and well-being. These existing frameworks drove the research questions and preliminary data coding. Themes such as autonomy, competence, and relatedness were derived from this theory to answer the research questions about the well-being of female professional musicians. The analytic framework also incorporated women’s experiences to explore how the professional female musicians in our sample discussed gendered issues such as caregiving responsibilities and work-life balance. The questions were not gendered *per se*, allowing the codes to naturally emerge without being prompted.

We followed a procedure for coding themes applied to SDT based on Raabe and Readdy (2016). First, after familiarizing themselves with the transcripts, all authors (serving as coders) independently highlighted relevant sections and assigned initial descriptive codes aligned with existing SDT literature. Next, each author independently grouped related data into 28 lower-order themes. The authors reviewed these preliminary themes independently, offering perspectives and making adjustments as necessary. Through an iterative process involving feedback from all authors, consensus was reached on the themes and codes that best represented participant experiences. In the first meeting, the 28 themes were consolidated into 19 based on their importance and how well they captured key participant ideas. In a second meeting, the 19 themes were either merged due to similarity, kept unchanged, or eliminated if they were mentioned by fewer than 30% of the sample. Saturation was achieved when no new themes emerged, resulting in a final set of 10 unique and significant themes. Subsequently, all authors independently confirmed that the themes accurately reflected the participants’ experiences. Finally, the authors selected participant quotes that best illustrated each theme and their experiences.

Then, the themes were refined inductively according to the participants’ narratives and experiences (Fereday and Muir-Cochrane, 2006). The inductive approach became prominent when refining the broad themes. As participants’ narratives were analyzed, new insights and themes emerged directly from the data rather than being strictly aligned with the predefined theory. This iterative process involved reviewing the participants’ experiences, allowing new codes and themes to emerge, which were not necessarily predicted by the theory.

In sum, this research used a rigorous inductive-deductive approach. Transcripts were coded for themes and sub-themes based on the participants’ narratives. These codes were organized to reflect both the prevalence (number of participants) and the depth of discussion surrounding each theme. While Table 2 provides a summary of the frequency of participants who mentioned each theme, the narrative analysis presents the contextual richness of the data.

**Table 2:** Themes and subthemes drawn from the thematic analysis.

Overarching themes	Sub-themes	% (N)	Effect on well-being
3.1. Psychological needs	3.1.1. Autonomy	–	
	*3.1.1.1. Satisfaction*	68.75 (11)	Positive
	*3.1.1.2. Thwarting*	62.50 (10)	Negative
	3.1.2. Competence	–	
	*3.1.2.1. Satisfaction*	50.00 (8)	Positive
	*3.1.2.2. Thwarting*	68.75 (11)	Negative
	3.1.3. Relatedness	–	
	*3.1.3.1. Satisfaction*	75.00 (12)	Positive
	*3.1.3.2. Thwarting*	50.00 (8)	Negative
3.2. Intrinsic motivation	3.2.1. Love of music	68.75 (11)	Positive
	3.2.2. Sense of transcendence	31.25 (5)	Positive
3.3. Challenges of being a female musician	3.3.1. Work-lifebalance	81.25 (13)	Negative
	3.3.2. Resilience	68.75 (11)	Positive

*N = 16; % represents the percentages of participants in the sample who mentioned each theme, with the N in parentheses. The number beside each theme and sub-theme relates to the corresponding theme in the results section.*

## 3 Results and discussion

As shown in Table 2, the analysis of interviews with the 16 professional female musicians uncovered three key themes and 10 sub-themes related to their well-being. The three key themes were: (1) Basic psychological needs for autonomy, competence and relatedness; (2) Sustained intrinsic motivation; and (3) Challenges of being a woman in music performance.

Table 2 presents a summary of the key themes and sub-themes, the number of participants who contributed to each sub-theme, and the overall positive or negative effects each of these subthemes had on the participants’ level of well-being.

### 3.1 Psychological needs for autonomy, competence, and relatedness

Psychological needs were identified as one of the overarching themes. As highlighted in the first section of Table 2, psychological needs encompassed six sub-themes: autonomy satisfaction and thwarting, competence autonomy satisfaction and thwarting, and relatedness autonomy satisfaction and thwarting.

#### 3.1.1 Need for autonomy

As can be seen in section 3.1.1 of Table 2, 69% of musicians mentioned the need for autonomy, such as a constantly renewed choice of staying in music and a greater opportunity to make choices as career advances, and a sense of control over their choices as essential to their well-being. Over 60% also mentioned some need thwarting situations, such as being dependent on contracts and callbacks, as a threat to their well-being.

##### 3.1.1.1 Autonomy satisfaction

The interviewees agreed that the facets of autonomy in their careers, such as a sense of choice and control over one’s own career, were crucial for their well-being. Most participants mentioned that they were musicians by choice, and if they were allowed to change careers, they would stay in music.

There are many people who have influenced me, and I think they make it so that… I think it’s a choice I constantly make, to keep doing what I do. I think it has a lot to do with colleagues, people I also see and admire. I think there is always a way to reinforce that choice by seeing the people around me. (Part. 11).

Some participants even perceived this choice of field as a calling, a vocation:

It chose me, one way, because I played instruments from the time I was four. We think “Oh maybe I should do something else. Is this worth it? Is this gonna work? Am I gonna make a living?” There’s a lot of doubt. But […] my inner self was telling me I had to do it. Because I did not have anybody telling me what to do anymore. (Part. 15).

The interviewees’ experiences underscore the critical role that autonomy plays in their professional lives, emphasizing the freedom to make independent career choices as a significant factor in their overall satisfaction. Autonomy as a core psychological need is particularly relevant in this context (Evans, 2015; Ryan and Deci, 2017). For musicians, the ability to choose their career paths freely contributes to their sense of purpose. Participants’ continuous choice to stay in music, driven by the influence and admiration of colleagues, aligns with findings in music psychology that suggest autonomy-supportive environments foster greater well-being among musicians (Bonneville-Roussy et al., 2020). Similarly, music being a calling reflects the concept of eudaimonic well-being (Ryff and Keyes, 1995), where personal growth and pursuing one’s true potential are central to one’s professional life (MacDonald et al., 2017). This continued sense of choice reinforces the musicians’ commitment to their field despite career uncertainties.

##### 3.1.1.2 Autonomy thwarting

The results showed that the lack of autonomy in the musicians’ careers negatively impacted their well-being. Many participants mentioned that being dependent on contracts and callbacks made them feel insecure and constrained in their professional lives.

So I do not know if, well, it must be like that for pretty much everyone in freelancing, but you know when your phone or your email, when you do not get as many job offers, whether you like it or not, you know it makes you question yourself a lot and then, well, psychologically it messes with your head. (Part. 6).

One participant felt discouraged as she experienced job insecurity despite all the efforts and achievements she had made throughout her career.

I think we put in more hours, you know, the hours I practice or rehearse, or the courses I take, we put in an enormous amount of hours, and yet… I do not consider that I have a profession or a position that allows me to make a living, you know. So yes, I manage really well, because I’m freelancing and I work a bit everywhere, but having a job that guarantees a certain security, I have not achieved that yet. […] I find it discouraging. (Part. 9).

Musicians also observed that the inability to refuse work or take career breaks led to substantial stress and frustration.

Well, I need to learn to say no. And that’s what I’m doing, I’m learning to say no. I’m taking fewer and fewer contracts. […] If I say no, they will not like me anymore, they will not call me anymore. (Part. 3).

Some musicians pointed out that auditions demanded considerable effort but provided minimal control over the outcomes, resulting in feelings of helplessness and discontent.

And it’s not the kind of field where, even if you succeed 1,000 times, it all rests on an audition. So you tell yourself, I cannot even be sure that, you know, by doing super rigorous training or studying more, by maintaining good contacts, you know, that’s not even what really determines if you’ll get the job. (Part. 14).

The findings indicated that a lack of autonomy significantly impacted the well-being of musicians. The feeling of a lack of control reflects broader issues at work, where job insecurity can lead to significant psychological stress (Vander Elst et al., 2014). This aligns with findings from studies on freelance musicians, which highlight the precarious nature of their work and its impact on mental health (Gross et al., 2018; Musgrave and Gross, 2020). The feeling of discouragement and lack of professional stability is common in the music industry, where the balance between effort and reward often appears skewed (Gross et al., 2018; Loveday et al., 2023). A study conducted with musicians who had utilized mental health services revealed that more than three-quarters of them reported high or overwhelming financial stress and perceived lack of choice in accepting contracts, which was also associated with higher levels of self-reported depression and anxiety (Berg et al., 2022). The inability to secure a stable position despite extensive practice and training exacerbates frustration. Despite the personal cost, this pressure to accept all available work is a well-documented issue in freelance professions, where job scarcity and competition are high (Bennett, 2016). Furthermore, some musicians pointed out that auditions demanded considerable effort but provided minimal control over the outcomes, resulting in feelings of helplessness. This lack of control over career advancement is a critical issue, contributing to the overall stress and dissatisfaction experienced by some musicians.

#### 3.1.2 Need for competence

Section 3.1.2 of Table 2 shows that while 50% of musicians reported competence satisfaction, 69% of them stated elements of competence thwarting. Musicians conveyed that their need for competence was fulfilled daily through experiences of mastery, learning, and improvement of their musical skills. This satisfaction was associated with enhanced feelings of well-being. Conversely, insufficient preparation or time to fully master a repertoire or a new skill was cited as a source of stress and feelings of inadequacy (Vellacott and Ballantyne, 2022).

##### 3.1.2.1 Competence satisfaction

Many musicians indicated that music was their primary area of competence and found it stimulating to engage in continuous learning and challenges.

You know what I mean, I move forward with my backpack, and my backpack keeps filling up. But it’s like Hermione’s bag in Harry Potter. It has no bottom, you know. I can just keep adding things and learning, developing new knowledge, developing new skills, always learning more. (Part. 13).

They also mentioned experiencing greater enjoyment of performances when mastery was achieved through dedicated practice.

When you feel like you have really reached… like… you have found something special, you have also reached a good level of preparation. Then, sometimes it feels like everything is going well, I do not know, you get on stage, you feel good […]. Those concerts are really fun. (Part. 9).

The perspective that music is the primary competence area for many musicians indicates that continuous learning and skill development are crucial for musicians’ professional growth and personal satisfaction (Manturzewska, 1990). Musicians mentioned the deep satisfaction of achieving a high level of skill (Vellacott and Ballantyne, 2022). Araújo and Hein (2019) have found that more time spent practicing daily is linked to experiencing flow, indicating that flow might enhance one’s commitment to daily practice. This phenomenon is supported by flow and peak performance theories, which suggest that high levels of skill and preparation lead to highly rewarding and enjoyable experiences (Seligman and Csikszentmihalyi, 2000). The satisfaction derived from achieving mastery contributes to a positive feedback loop, reinforcing the musicians’ commitment to continuous practice and improvement (Bonneville-Roussy and Bouffard, 2015).

##### 3.1.2.2 Competence thwarting

Musicians also reported that insufficient practice time led to stress and being caught up in the music’s technical aspects, hindering their sense of enjoyment.

Because sometimes I feel like I have not practiced enough, I’m stressed, uh, I’m not mentally available… to what’s around me. Uh… yes, it creates stress for me. Sometimes, it causes insomnia for me, sometimes. (Part. 2).

This is consistent with existing research, which suggests that inadequate preparation can lead to heightened stress and decreased performance quality (Kenny and Ackermann, 2016; Vellacott and Ballantyne, 2022).

#### 3.1.3 Need for relatedness

In this study, the need for relatedness emerged as a major theme among female musicians, with 75% of them reporting elements of relatedness satisfaction in their answers and 50% of relatedness frustration (section 3.1.3 of Table 2). They all noted that most of their friends were fellow musicians and that the music industry felt like a family. However, it was a significant source of stress when relationships were conflictual.

##### 3.1.3.1 Relatedness satisfaction

Some musicians have mentioned that music, especially when played in orchestras or ensembles, has the power to bring people together.

I’m attached to people, I’m attached to the beauty of how they coexist. I find an orchestra really amazing. I have a fascination for all the cohesion that can exist, and how people come together and do their best, collectively, to give the best result. It’s like… It does not happen often in society. (Part. 12).

Another theme highlighted by the participants was that relationships with colleagues offered mutual support and long-term friendships, which were sources of happiness and fulfillment.

I find that it allows for the development of beautiful relationships. […] So I have a lot of friends that I’ve met, and I think that, it’s a really nice environment. […] And I found it interesting because, I think that, we have friendships that we have kept for a long time. And you know, I’m always happy to see, you know, like sometimes I have a gig and it’s like, “oh,” you know, “you are here.” And you know, it’s fun to see each other like that. (Part. 14).

Finally, the musicians identified the importance of sharing music with family, friends, and the general public as a source of well-being for both themselves and others.

Regarding family, part. 5 said:

It can have a positive impact because, you know, it also brings people together. You know, that having events and concerts, like my family is super happy to come and hear me, and it creates events where we gather afterward, we go eat together.

Part. 1 added in relation to friends:

It’s super nice to give that to friends, to be able to offer music, it’s something we do […] But in my personal life, offering concerts to friends, going to their living rooms, it’s something we really enjoy doing, which is very, which is very rewarding. There is a certain power in music, to use it to bring joy and have good times.

One musician mentioned the enjoyment she gets from passing on her knowledge to younger musicians as a mentor.

I feel like I have enough expertise and enough knowledge that I just love to share it with younger musicians, as a mentor or as a coach. And I want to share my pleasure. I want to share my pleasure with everyone. And help people, especially help students to find that also I can go into, because I feel very competent. (Part. 15).

Many musicians mentioned that playing music fosters a sense of togetherness among musicians, relatives, and people outside of the music industry. This shared effort reflects findings that participating in ensembles strengthens social connections and a sense of belonging among people (Creech et al., 2013). Musicians also highlighted the joy of developing enduring friendships through music and the pleasure of reuniting with colleagues during performances. This observation aligns with research suggesting that strong interpersonal relationships in professional settings greatly enhance well-being and job satisfaction (Kansky, 2017), as well as the role of music in strengthening social bonds and enhancing communal joy (Creech et al., 2013).

##### 3.1.3.2 Relatedness thwarting

Almost all musicians mentioned that the music industry could also threaten their need for relatedness, leading to decreased well-being. Since the community of musicians is close-knit, they could not avoid toxic relationships.

On a social level, the circle definitely becomes the people we work with because we have all studied together and worked together. […]. And there’s like… it’s super hermetic. […] Interviewer: And is that positive or negative? Participant: Both. I think it’s positive: it creates tight bonds. But the flip side is that everyone knows each other and what we do is so intimate that it can be both positive and negative. It depends on who. (Part. 16).

For instance, one musician mentioned that she had a strained relationship with a colleague in her orchestra, with whom she had to interact several times a week. This strained relationship led to increased stress and decreased satisfaction during rehearsals.

Regarding being the section lead in an orchestra, one participant said:

Let us say the last concert, I was the one in front, so it was kind of me who had to handle… everyone’s emotions. […] Of course, when I was in that situation last time, well… I cannot say it contributed to my happiness (laughs). It’s… it’s more stressful, it’s less enjoyable. […] It’s always little comments like that, which makes relationships harder and it’s also stressful. (Part. 7).

One musician mentioned that the lack of preparation from colleagues before a performance could be unsettling, affecting her well-being as a result.

For me, if I’m sitting with someone… who has not practiced well, who does not know what they are doing or what… it’s really distracting to be sitting with someone who is not well-prepared. (Part. 9).

One participant mentioned that being part of an ensemble could lead to feelings of anonymity and a lack of recognition.

For example, again, as a violinist, we are part of a violin section. So, it’s very anonymous. It can be challenging sometimes, I mean, I would not be there. […] I practiced 30 h to be able to play that, but I would not be there and no one would notice. (Part. 1).

The need for meaningful connections with others is thwarted when musicians feel that they have unhealthy relationships with their colleagues, or that these connections are meaningless. In our study, musicians described how their social circle is predominantly composed of colleagues they have studied and worked with, creating strong bonds and potential for negative interactions due to the intimate and hermetic nature of the community. Depending on the individuals involved, this duality can lead to positive and negative experiences (Ascenso et al., 2022). Musicians also described the challenge of feeling unnoticed despite significant effort and preparation. The sense of anonymity within a larger group can diminish a musician’s sense of worth and satisfaction (Kenny et al., 2014).

### 3.2 Intrinsic motivation

Intrinsic motivation refers to engaging in an activity for the inherent satisfaction and pleasure derived from the activity, rather than for external rewards or pressures (Evans, 2015, 2023). All musicians mentioned forms of intrinsic motivation in their interviews, highlighting that being intrinsically motivated was a major source of well-being. Two sub-themes were prevalent with regards to intrinsic motivation: the inner love for music, reported by 69% of the sample, and a transcendental experience (31%, see section 3.2 of Table 2).

#### 3.2.1 Love of music

Almost all musicians expressed a deep love for their craft. Specifically, many mentioned that practice was inherently enjoyable and often led to feel-good moments.

When we practice, we cannot think about anything else. So it’s a, it’s like a moment… a very… very good moment in a day. There’s not much during the day that will bother us. […] We’re in a pretty, pretty calm zone. (Part. 4).

Others said they used practice as a form of self-regulation to improve their psychological well-being.

And well, music in itself, I think, is something therapeutic in life, so, uh, when I’m stressed, or when I’m sad, or whatever, well, often I take out my [instrument] and, uh… it makes me feel better. (Laughs). (Part. 7).

One musician mentioned that music was so enjoyable that it felt more like a hobby than a career to her.

I really feel like it’s more of a hobby, something I would do, and I do not feel like it’s a chore. (Part. 7).

One musician referred to a sense of flow or of ‘being in the zone’ while playing.

It soothes the soul at times. And even, you know, there are moments, like when we talk about flow, there are moments where everything feels right technically, and with your instrumental comfort. […] It’s in those moments that you really feel like saying, ‘it’s all worth it for moments like this. (Part. 14).

According to SDT, intrinsic motivation arises when engaging in an activity for its inherent interest or pleasure (Deci and Ryan, 1985; Ryan and Deci, 2017). Musicians often exhibit intrinsic motivation through their love for music and the enjoyment they derive from playing their instruments. This intrinsic motivation leads to increased practice time (MacIntyre et al., 2018), higher quality practice sessions, and a greater preference for challenging tasks (Evans and Bonneville-Roussy, 2016). Additionally, intrinsic motivation helps musicians build resilience against the challenges of pursuing a musical career, and increase musicians’ sense of flow (Habe et al., 2019; Schnare et al., 2012).

#### 3.2.2 Sense of transcendence

Musicians referred to recurring experiences where they felt a profound sense of elevation and transformation while engaging with music. These moments go beyond the ordinary, often evoking intense emotions and a profound sense of well-being.

Sometimes I play pieces, and I’m like, “Wow, God exists,” you know. The piece is so fantastic, you think, “Wow, this is not human, it’s not a human who wrote this, it comes directly from divine inspiration,” you know. (Part. 3).

One musician described that her love of music was almost transcendental:

There are emotions that I feel sometimes when I’m playing music that I do not ever feel at other times. Like emotions that I did not know existed. I can feel them when I’m playing music. So it’s like a healing process also. (Part. 15).

Music evokes emotions and sensations that are rarely found elsewhere. This aligns with existing literature, highlighting music’s ability to evoke peak experiences and facilitate a sense of connection to something greater than oneself (Gabrielsson, 2011). When individuals fully immerse themselves in music, they can experience an expanded sense of self (Bernard, 2009). Musicians often perceive music as something greater than themselves, allowing them to delve deeper into their emotions (Hruska and Bonneville-Roussy, 2022; Scharff, 2018; Sergeant and Himonides, 2014). This deep emotional connection contributes to their enjoyment and pleasure when playing their instruments, serving as a source of intrinsic motivation.

### 3.3 Challenges of being a female musician

Although we did not specifically inquire about the challenges faced by female musicians, most women in the study highlighted them. The primary sub-theme identified was the difficulty maintaining a work-life balance, stated by 81% of the sample (section 3.3.1 of Table 2), negatively impacting their well-being. Interestingly, another sub-theme that emerged was that, for some women, these challenges fostered personal growth and resilience, ultimately enhancing their well-being over time (reported by 69%, section 3.3.2 of Table 2).

#### 3.3.1 Work-life balance

The necessity of maintaining a work-life balance was a major concern among our participants. Juggling atypical schedules and working while others were off created family and social life issues.

Sometimes I find it difficult to be a very, very, very high-level musician and have a balanced life with family, with children, especially for women. […] It’s really a lifestyle where you have to make decisions based on what you want and what you need for your life balance. And sometimes it works, sometimes it does not. So it has a significant impact on our personal life. On our family life. On our colleagues. (Part. 16).

Many women questioned motherhood and family life as a result of their career choice, with several mentioning they had delayed having children because the demands of being an early-career musician were incompatible with the constraints of a young family.

And you know, even just making life decisions. Like important decisions. For instance, whether we can have children. (Part. 14).

There are so many things to consider. In the sense that I also want to be a mom at some point. So it will not be my peak time for [instrument]. And it’s a project that will happen soon, I hope. So let us say we give it a year for my peak [laughs]. (Part. 10).

Additionally, many women mentioned having to choose between spending more time with their children and life partners or playing more music.

So sometimes it… bothers me a bit to say, “Well, listen, my dear, I have to go practice, and… that’s life.” I would spend time with [family], but you know, I do not really have a choice. (Part. 8).

Sometimes my daughter says “You’re out too much at night.” For concerts. […] She feels abandoned. I mean, not abandoned, but you know, like she feels like I’m sometimes out too much. At certain periods of the years even my son sometimes in very busy periods of concerts, he’ll be like, “Mommy you are not here.” So there’s a drawback of being a performing musician for family life. (Part. 15).

Some musicians said that having such an atypical schedule made it challenging to maintain a social network outside of music.

And of course, the fact that our schedules are atypical, we work when others have free time. Like, friendships, for example, I have many friends who are teachers. So it’s not always easy to be able to see each other because I work evenings and weekends. They work during the day, on weekdays. (Part. 13).

Our participants faced significant challenges in balancing work and life, particularly when deciding whether to have children. Female musicians, in particular, must manage family responsibilities alongside their careers (McDowall et al., 2019). As primary caregivers, women have the additional burden of organizing childcare, which is especially challenging due to the unconventional hours musicians work, such as weekends and evenings when schools and daycares are typically closed. This scheduling conflict results in less time spent with their children than desired. Additionally, maternity leave and childcare responsibilities are frequently viewed negatively, disadvantaging women (Curtis, 2013). Musicians also struggle to spend time with their partners and friends. These findings align with the literature indicating that work-life balance is one of the most significant challenges musicians face (Bartleet et al., 2020). The competition between professional demands and family life negatively impacts musicians’ well-being (Musgrave, 2023).

#### 3.3.2 Resilience

Resilience is a critical attribute for professional musicians, enabling them to navigate their careers’ various demands and pressures. The stress and challenges associated with musical performance are well-documented in the literature, and these elements are seen not only as obstacles but also as opportunities for personal growth and development. Resilience in this context involves the ability to manage performance anxiety, physical strain, and the high expectations placed upon musicians (Kegelaers et al., 2021).

In this study, some participants said that being a professional musician comes with challenges that allow them to grow as a person.

Well, of course, it’s stressful. […] But it makes you work on yourself. I think it’s not negative, and wanting to do this job means you have to learn to juggle that aspect, the performance stress and the physical strain too. […] But after that, you learn to work so that it’s as painless as possible. And easier. (Part. 16).

For example, a musician explained that she had to become more self-compassionate to deal with the high expectations of a career in music.

Maintaining the technical level, to always be at the top of your form, it’s complicated. It’s complicated. I have a young child… and there are many factors. I have students to manage, I have a lot of things going on. So it definitely requires… a lot of self-compassion. (Part. 8).

Other participants said that the atypical working hours led them to develop organizational skills and strategies that allowed them to make the most of their practice time.

You know, unlike others who are not in this situation, for example, who have more free time in the evenings and weekends, I know that I have this in the afternoon, I need to learn… ‘this’ sheet music, you know. I have to do my work with the utmost concentration. So I try to put technologies aside, so I will not be disturbed […] (Part. 2).

Musicians also said that music practice enabled them to develop and maintain their cognitive and physical abilities.

I find an extraordinary benefit for the development of my brain, the shaping of my brain. Uh, maintaining, maintaining gains, both in terms of reflexes, mental quickness, and good mood. (Part. 4).

Despite the numerous challenges our participants faced, playing music enabled them to grow and strengthened their ability to overcome difficulties. They built resilience by fostering self-compassion, developing organizational and time management skills, and maintaining their focus(Kansky, 2017). One participant noted the cognitive benefits of playing music, which has been shown to positively impact various cognitive functions, including memory (Bruhn, 2002). These resilience factors positively influence musicians’ well-being, as resilience is negatively associated with mental health issues such as anxiety and depression (Kegelaers et al., 2021).

## 4 Conclusion

This study provides novel insights into the well-being of professional female musicians through the lens of SDT. While previous research has predominantly focused on structural barriers and negative outcomes, this study takes a more holistic approach, integrating both positive and negative aspects of well-being in the lived experiences of female musicians. Our findings highlight the critical role of psychological needs satisfaction in shaping their well-being. Importantly, this study is among the first to qualitatively explore how the unique challenges faced by women in music and psychological needs satisfaction converge, offering a richer understanding of how female musicians experience them. By addressing this gap, we contribute to a more nuanced understanding of well-being in the music industry and propose actionable insights for creating more supportive environments for women in high-performance domains.

The findings emphasized the critical importance of satisfying psychological needs for autonomy, competence, and relatedness, as well as intrinsic motivation, in enhancing the well-being of these musicians. In general, our results have highlighted that female musicians report more positive than negative effects of being a musician on their well-being. In terms of the PERMA model of well-being, our results revealed that musicians’ musical environment provided them with positive emotions, meaningful relationships and engagement with others and music (Ascenso et al., 2017, 2018; Bonneville-Roussy and Vallerand, 2020). This led to a sense of life purpose and deep satisfaction with their craft. However, some aspects of the music industry also posed challenges to their well-being. In particular, women in music seem to still experience greater work-life balance issues. Our study goes beyond confirming SDT’s relevance to musicians’ well-being by offering new insights into how female musicians navigate gender challenges that affect their psychological needs and well-being. These findings highlight the importance of addressing gender-specific barriers to promote well-being in high-performance fields like music.

Participants highlighted that their sense of autonomy was critical to their well-being because it enabled them to feel in control of their career paths. However, the lack of autonomy created stress and insecurity. Regarding competence, musicians stated that their everyday experiences of mastery, learning, and skill advancement were critical to their well-being. The deep satisfaction of obtaining high proficiency levels via dedicated practice was linked with increased well-being. Conversely, insufficient practice time caused stress, reducing musicians’ enjoyment and perception of competence. Nearly all participants stated that their need for relatedness was met in music, which fostered a sense of family and mutual support. However, the close-knit structure of the music community presented difficulties, as conflicts within these connections were significant sources of stress.

Furthermore, the often isolating character of high-performance environments led to feelings of anonymity, reducing musicians’ sense of fulfillment. The musicians’ well-being was also influenced by intrinsic motivation, or engaging in an activity for its inherent satisfaction. A prominent element of the current study was a great love of music. The participants described moments of transcendence when performing music, with a feeling that music was greater than themselves, which offered profound emotional and psychological satisfaction.

The gender-specific challenges faced by professional female musicians emerged as a significant, though not explicitly inquired about, aspect of this study. Many participants highlighted issues related to work-life balance, caregiving responsibilities, and professional instability—challenges that disproportionately affect women in high-performance fields like music. These findings align with existing literature, which underscores that female musicians often face greater difficulties in maintaining career momentum due to irregular work schedules, the need for extended leaves, and societal expectations around caregiving roles (Hruska and Bonneville-Roussy, 2022; McDowall et al., 2019). Without sufficient institutional or structural support, these challenges can undermine not only the professional advancement of female musicians but also their psychological well-being (Ascenso et al., 2017). Despite these problems, some participants reported that their experiences boosted their personal growth and resilience, allowing them to better manage the pressures of their careers. Although the study did not include a specific gender-focused question, the natural emergence of these themes underscores the importance of addressing gender in discussions of well-being and career satisfaction within the music industry (Scharff, 2018; Smith and Hendricks, 2022).

### 4.1 Limitations

This study provides valuable insights into the well-being of professional female musicians, but several limitations should be noted. The small sample size of 16 participants from Canadian classical orchestras may limit the generalizability of the findings. Future research should involve larger and more diverse samples. Additional data collection methods, such as direct observations or longitudinal studies, could help validate the results of this study. Additionally, the study did not explore external factors like institutional policies, cultural differences, or socioeconomic status. Finally, the cross-sectional design captures experiences at a single point in time, limiting insights into causality or changes over time. Longitudinal studies could offer more robust insights into how well-being and psychological needs evolve throughout musicians’ careers.

### 4.2 Implications and future research

In sum, professional musicians, particularly women in this field and other performance domains, would benefit from environments that satisfy the basic psychological needs for autonomy, competence, and relatedness. Although the industry has made significant progress in addressing systemic well-being issues, these psychological needs are still not fully met. Since satisfying these needs is a crucial predictor of intrinsic motivation and well-being, greater awareness of these issues is necessary. Ensuring that environments within the music industry support the psychological needs of autonomy, competence, and relatedness can significantly enhance musicians’ well-being and professional satisfaction. Additionally, female musicians face gender-specific challenges and often bear the primary responsibility for childcare, further constraining their well-being. Addressing these unique challenges female musicians face, such as work-life balance and the need for career stability, is crucial. Future research should address these concerns.

## Data Availability

The raw data supporting the conclusions of this article will be made available by the authors, without undue reservation.
